# A Novel Transformers Fault Diagnosis Method Based on Probabilistic Neural Network and Bio-Inspired Optimizer

**DOI:** 10.3390/s21113623

**Published:** 2021-05-23

**Authors:** Lingyu Tao, Xiaohui Yang, Yichen Zhou, Li Yang

**Affiliations:** 1 College of Information Engineering, Nanchang University, Nanchang 330031, China; 6104118095@email.ncu.edu.cn (L.T.); yangliedu@163.com (L.Y.); 2 College of Qianhu, Nanchang University, Nanchang 330031, China; 5701118215@email.ncu.edu.cn

**Keywords:** improved salp swarm algorithm, sine cosine algorithm, probabilistic neural network, disruption operator, power transformer, fault diagnosis

## Abstract

Since it is difficult for the traditional fault diagnosis method based on dissolved gas analysis (DGA) to meet today’s engineering needs in terms of diagnostic accuracy and stability, this paper proposes an artificial intelligence fault diagnosis method based on a probabilistic neural network (PNN) and bio-inspired optimizer. The PNN is used as the basic classifier of the fault diagnosis model, and the bio-inspired optimizer, improved salp swarm algorithm (ISSA), is used to optimize the hidden layer smoothing factor of PNN, which stably improves the classification performance of PNN. Compared with the traditional SSA, the sine cosine algorithm (SCA) and disruption operator are introduced in ISSA, which effectively improves the exploration capability and convergence speed. To verify the engineering applicability of the proposed method, the ISSA-PNN model was developed and tested using sensor data provided by Jiangxi Province Power Supply Company. In addition, the method is compared with machine learning methods such as support vector machine (SVM), back propagation neural network (BPNN), multi-layer perceptron (MLP), and traditional fault diagnosis methods such as the international electrotechnical commission (IEC) ratio method. The results show that the proposed method has a strong learning ability for complex fault data and has advantages in accuracy and robustness compared to other methods.

## 1. Introduction

Oil-immersed power transformers are among the most expensive and essential pieces of equipment in power systems [1,2,3]. During operation, oil-immersed transformers are subjected to various stresses, such as electrical, thermal, chemical, and mechanical stresses, which can lead to the aging and deterioration of their insulation. In addition, the insulation degradation or moisture generated by the external environment can accelerate the aging process, reduce the dielectric strength, and lower the partial discharge initiation voltage. Insulation defects are the most common cause of failure in excitation transformers and directly affect the reliability of the equipment [4,5]. In today’s increasingly large power demand, if a power transformer fails, it will likely cause an interruption of power supply to the energy system and bring significant economic losses. Therefore, being able to quickly and accurately diagnose the type of faults during transformer operation has become an important issue in promoting the smart grid process.

Currently, the dissolved gas analysis (DGA) method has been widely used in the fault diagnosis of oil-immersed transformers [6,7]. The occurrence of mechanical, electrical, and thermal faults in an oil-immersed power transformer leads to the degradation of the insulating oil and the increase in some gases. The causes of gas generation include corona (partial discharge), low energy sparks, arcing, cellulose overheating, and insulation overheating. In this case, gases such as carbon monoxide (CO), carbon dioxide (CO${}_{2}$), hydrogen (H${}_{2}$), methane (CH${}_{4}$), ethane (C${}_{2}$H${}_{6}$), ethylene (C${}_{2}$H${}_{4}$), and acetylene (C${}_{2}$H${}_{2}$) are dissolved in the oil in different proportions. Although the oil contains oxygen (O${}_{2}$) and nitrogen (N${}_{2}$), they enter the transformer from outside and are not related to the degradation of the insulating oil [8]. Therefore, through intelligent sensors and in order to obtain dissolved gas data in the oil and analyze it, we can achieve a real-time view of the operating conditions of the transformer to quickly and efficiently check the internal hidden problems and faults of the transformer [9,10]. Currently, the classical fault diagnosis methods based on DGA data include Doernenburg [11], Rogers [12], IEC 599 [13] and IEC 60599 [14,15]. They attempt to study the hydrogen (H${}_{2}$), methane (CH${}_{4}$), ethane (C${}_{2}$H${}_{6}$), ethylene (C${}_{2}$H${}_{4}$), and acetylene (C${}_{2}$H${}_{2}$) gas concentrations and the relationships between them, and then evaluate the transformer operating conditions according to various pre-defined thresholds [16].

Although the methods above are easy to apply, they require a large amount of engineering experience, are not sufficiently generalized, and their fault detection accuracy is limited. Thus, they may not be reliable enough for predicting fault types [17]. To improve the fault diagnosis accuracy to meet the current industrial demand, scholars in related fields have combined traditional fault diagnosis methods with emerging artificial intelligence technologies to improve the accuracy of fault diagnosis significantly. A series of classical statistical learning methods such as hidden Markov models (HMM) [18], support vector machine (SVM) [19], k-nearest neighbor (KNN) [20], etc., are applied to process DGA data to improve the accuracy of fault diagnosis. With the continuous development of data-driven technologies, new machine learning methods with better performance are combined with traditional fault diagnosis methods to achieve excellent diagnostic results. Wang et al. [21] improved the traditional fault diagnosis method, i.e., the dielectric response method. Low-frequency dielectric parameters were first extracted using mixed-frequency excitation. Then, the extended Debye equivalent circuit parameters were determined using the cuckoo search (CS) optimization algorithm. Finally, the specific parameters were used for testing to establish a simulation model and obtain a recovery voltage curve. Compared with the traditional method, this method greatly reduces the test time. To cope with the high uncertainty and variability of DGA data, Kirkbas et al. [22] used the information-rich feature data set obtained by spectral techniques along with the common vector approach (CVA) for the fault mode identification of DGA data. The CVA-based method is well suited and has better fault diagnosis performance than the traditional SVM-PSO method, as demonstrated by examples. Jiang et al. [23] proposed lasso regression to build a multidimensional linear model of the selected features. The change point detection method based on lasso regression is based on the minimum days and standard deviation (SD) between the change point and fault time, which accurately reflects the location of the transformer fault in most cases. The method provides an effective method for dynamic fault prediction based on dissolved gas data with the advantages of robustness and no data training. The application of an artificial neural network (ANN) in the field of fault diagnosis of oil-immersed transformers [24] has led to a significant improvement in the reliability of diagnosis. Meanwhile, optimization schemes for a neural network are becoming more abundant and mature. Yang et al. [17] proposed a power transformer fault diagnosis system combining a polynomial logistic regression model and a back propagation neural network (BPNN) to determine the type of transformer faults by analyzing the dissolved gases in the transformer. The test results show that this intelligent fault diagnosis system’s recognition rate is about 10–30% higher than that of the single neural network or multi-neural network recognition system without a polynomial logistic regression model. Huang and Wang et al. [25] proposed a transformer fault diagnosis method based on the gray wolf optimization (GWO) algorithm to optimize the hybrid kernel function learning machine. The parameters of the hybrid kernel function can be optimized using the GWO algorithm. Simultaneously, the initial population parameters of the GWO algorithm are generated by using chaotic logistic mapping to avoid the adverse effects of overly fast convergence on the optimization results, which effectively improves the classification accuracy. Dai et al. [26] developed an oil-immersed fault diagnosis model based on a deep belief network (DBN) and compared the performance of the method with the support vector machine (SVM), back propagation neural network (BPNN), and ratio method. The results show that the method significantly improves the accuracy of power transformer fault diagnosis. Ou et al. [27] proposed a dynamic Adam and dropout-based deep neural network (DADDNN) for oil-immersed power transformer fault diagnosis. Ou et al. utilized the dropout technique to randomly reset some neurons to prevent overfitting and indirectly enhanced the information exchange between them.

It is clear that ANN has promising applications in fault diagnosis. There are many different types of ANN available for classification tasks. Probabilistic neural network (PNN), as a radial neural network, has a strong fault tolerance. PNN can converge to a Bayesian classifier as long as sufficient sample data are available, which is more efficient than other network models such as the multi-layer perceptron (MLP) and the back propagation neural network (BPNN). In addition, PNN has some advantages in solving multi-classification problems [28,29].

In this paper, PNN was chosen as the basis for the fault diagnosis classifier. Since the classification performance of PNN is easily affected by the smoothing factor (σ) of the hidden layer [30], the selection of the smoothing factor (σ) can make the network converge too quickly and lead to a significant decrease in classification accuracy. Therefore, we used the improved salp swarm algorithm (ISSA) optimized by the sine cosine algorithm (SCA) and disruption operator (D${}_{op}$) to filter the smoothing factor (σ). SCA introduces the update mechanism of SSA, which enhances the exploration capability and reduces the possibility of getting into the local optimum. The disruption operator (D${}_{op}$) was used to improve the population diversity and maintain the balance between exploration and exploitation processes. To verify whether the ISSA-PNN model is applicable to the field of oil-immersed transformer fault diagnosis, we compared it with traditional fault diagnosis methods and other intelligent algorithm models based on the same set of DGA data. The experimental results show that ISSA has a better performance on the DGA experimental data set than the traditional SSA. The ISSA-PNN method outperformed other methods in terms of accuracy, diagnosis efficiency, and robustness compared with other fault diagnosis methods.

The rest of the paper is organized as follows: Section 2 describes the proposed method. In Section 3, the transformer fault diagnosis model is described. Section 4 presents the experimental results, and Section 5 discusses them. Finally, the conclusion is drawn in Section 6.

## 2. The Proposed Method

In this section, we present the proposed fault diagnosis method for power transformers. We first introduce the salp swarm algorithm (SSA), the sine cosine algorithm (SCA), and then discuss the improved salp swarm algorithm (ISSA). Finally, the ISSA-based probabilistic neural network fault diagnosis model is described in detail.

### 2.1. Salp Swarm Algorithm

The salp swarm algorithm (SSA) is a novel intelligent optimization algorithm proposed by Seyedali Mirjalili et al. in 2017 [31]. The algorithm performs an optimization search process in the solution space by simulating the salps swarm’s predation behavior. In the deep sea, the salp group moves and feeds in a chain behavior. There are leaders and followers in the chain, and the leaders move towards the food and guide the followers to follow them. At each iteration, the leader performs global exploration while the followers fully explore locally. Compared with other algorithms, the iterative optimization-seeking mechanism of the SSA algorithm dramatically reduces the cases of falling into the local optimum.

The specific process of SSA can be divided into two steps, as follows.

**Step 1:** Initialization of SSA. Set the number of populations *N*, the spatial dimension *d*, the maximum number of iterations *T*, and initialize the salps population’s position by Equation (1):  

(1) $$X_{\left(N,d\right)}=rand\left(N,d\right)\cdot \left(ub-lb\right)+lb$$

where ub and lb denote the upper and lower bounds of the search space, respectively; the matrix represented by X(N,d) stores the bottle’s positions ascidian group.

**Step 2:** Position Update.

(1) The leader position is updated, as shown in Equation (2):

(2) $$X_{j}^{leader}=\left\{\begin{array}{c} F_{j}+c_{1}\left(\left(ub-lb\right)c_{2}+lb\right),c_{3}\geq 0.5\\ F_{j}-c_{1}\left(\left(ub-lb\right)c_{2}+lb\right),c_{3}<0.5 \end{array}\right.$$

where $X_{j}^{leader}$ and $F_{j}$ in Equation (2) represent the leader and food positions in the *j*th dimension, respectively. Since the position of the food/target is not clear in the actual iterative process, the salp’s position with the current optimal fitness value is set as the food position in each iteration process. $c_{1}$, $c_{2}$, $c_{3}$ are control parameters, where $c_{1}$ is the convergence factor in the algorithm, which is the essential control parameter in SSA and plays the role of balancing the global search and local exploitation capability, and its expression is:

(3) $$c_{1}=2e^{-{\left(\frac{4t}{T}\right)}^{2}}$$

where *t* represents the current number of iterations, and it can be seen that the convergence factor is a decreasing function from 2 to 0 during the iterative process. $c_{2}$ and $c_{3}$ are random numbers of [0,1], which are used to enhance $X_{j}^{leader}$ ’s randomness to improve the global search capability of the algorithm.

(2) The followers advance in a chain-like sequence by influencing each other between the individuals before and after them. Their displacements are following Newton’s law of motion, and the motion displacement of the followers can be expressed as

(4) $$X_{j}^{i}=\frac{1}{2}at^{2}+v_{0}\Delta t$$

where $X_{j}^{i}$ is the position of the *i*th follower in the *j*th dimension, and *a* is the acceleration and is calculated as

(5) $$a=\left(v_{final}-v_{0}\right)/\Delta t$$

where $v_{final}=\left(X_{j}^{i-1}-X_{j}^{i}\right)/\Delta t$, $X_{j}^{i-1}$ is the position of the i−1th salp in the *j*th dimensional space. Since $v_{0}=0$ and time *t* is the number of iterations, i.e., Δt=1 during the algorithm, Equation (4) can be expressed as

(6) $$X_{j}^{i}=\frac{X_{j}^{i}-X_{j}^{i-1}}{2}$$

### 2.2. The Sine and Cosine Algorithm

The SCA algorithm is a stochastic optimization algorithm that is highly flexible, simple in principle, easy to implement, and easily applied to optimization problems in different fields [32]. The optimization process of the sine cosine optimization algorithm can be divided into two phases: in the exploration phase, the optimization algorithm quickly finds a feasible region in the search space by combining a specific stochastic solution among all stochastic solutions; in the development phase, the stochastic solution will gradually change, and the speed of the change of the stochastic solution will be lower than that of the exploration phase.

In the sine cosine algorithm, the candidate solution is first randomly initialized. Then, the current solution is updated in each dimension according to the sine or cosine function combined with a random factor. The specific update equation is:   

(7) $$X_{j}^{t+1}=\left\{\begin{array}{c} X_{j}^{t}+r_{1}\cdot \sin\left(r_{2}\right)\cdot \left|r_{3}P_{j}^{t}-X_{j}^{t}\right|\;r_{4}>0.5\\ X_{j}^{t}+r_{1}\cdot \cos\left(r_{2}\right)\cdot \left|r_{3}P_{j}^{t}-X_{j}^{t}\right|\;r_{4}\leq 0.5 \end{array}\right.$$

In Equation (7), $X_{j}^{t}$ is the position of the *j*th dimension of the current individual in the *t*-th generation, $r_{2}$ is a random number from 0 to 2π, $r_{3}$ is a random number between 0 and 2, $r_{4}$ is a random number from 0 to 1, and $P_{j}^{t}$ denotes the position of the *j*th dimension of the optimal individual position at *t* iterations. $r_{1}$ can be expressed as

(8) $$r_{1}=a-t\frac{a}{T}$$

where *a* is a constant, *t* is the current number of iterations, and *T* is the maximum number of iterations. The value of $r_{1}$ decreases gradually with the iterative process, balancing the algorithm’s local exploitation and global search capability.

### 2.3. Improved Salp Swarm Algorithm

To improve the exploration and exploitation capabilities of SSA, we used SCA instead of the traditional follower position update mechanism in SSA and introduced a disruption operator ($D_{op}$) to increase the diversity of the salp population [33]. To achieve this goal, Liu et al. [34] gave the following equation to define the disruption operator:

(9) $$D_{op}=\left\{\begin{array}{c} D_{i,j}\cdot rand\left(-2,2\right)\;\;\;\;\;\;\;if\;D_{i,best}\geq 0.2\\ 1+D_{i,best}\cdot rand\left(-\frac{1}{20},\frac{1}{20}\right)\;\;otherwise \end{array}\right.$$

In Equation (9), $D_{i,j}$ represents the distance between the *i*th solution and the *j*th nearest solution, and $D_{i,best}$ describes the distance between the *i*th solution and the best solution.

The initialization process of ISSA and the leader update mechanism are consistent with the traditional SSA. However, the update mechanism of followers is not the same as SSA. At this time, the update method of SCA is selected instead of the traditional SSA update method, that is, using Equation (7) for the position update of followers.

The disruption operator is introduced after the end of the position update, and to reduce the computation time in this phase, the disruption operator is used as shown in Equation (10):

(10) $$X^{\prime }=\left\{\begin{array}{c} X\cdot D_{op}\;\;\delta_{0}>0.5\\ \;\;X\;\;\;\;\;\;\delta_{0}\leq 0.5 \end{array}\right.$$

where $X^{\prime }$ represents the updated population of salps using the disruption operator. It can be seen from Equation(10) that the disruption operator is used to diversify the salp population only when the random number $\delta_{0}$ is greater than 0.5. The pseudo-code of SCA-SSA is shown in Algorithm 1.
**Algorithm 1** Improved salp swarm algorithm.1:**Initialization parameters:** population size *N*, dimension *d*, maximum number of iterations *T*.2:Generate the initial population *X* by Equation (1);3:Calculate the fitness value for each individual;4:**while**t<=T**do**5:    Update $c_{1}$ by Equation (3) and $r_{1}$ by Equation (8);6:    **for** i=1:n **do**7:        **if** Xi(leader) **then**8:           Update random numbers $c_{2}$ and $c_{3}$;9:           Update the position of the leader salp as in Equation (2);10:        **else**11:           Update random numbers $r_{2}$, $r_{3}$ and $r_{4}$;12:           Update the position of the follower salp as in Equation (7);13:        **end if**14:        Calculation $D_{op}$ using Equation (9);15:    **end for**16:    **if** $\delta_{0}>0.5$ **then**17:        $X=X\cdot D_{op}$;18:    **end if**19:    Set t=t+1;20:**end while****Output:** Best classification and predication results.

### 2.4. Probabilistic Neural Network

A probabilistic neural network (PNN) is a radial basis network that belongs to a feed-forward kind network. It has the following advantages: simple learning process, fast training speed, more accurate classification, good fault tolerance, etc. In essence, it belongs to a supervised network classifier based on the Bayesian minimum risk criterion.

Probabilistic neural networks generally have four layers: input layer; pattern layer; summation layer; and output layer. Among them, the pattern layer is connected to the input layer by connecting weights, calculating the degree of matching between the input feature vector and each pattern in the training set, that is, the similarity, and feeding its distance into a Gaussian function to obtain the output of the pattern layer. The output of each pattern unit is as follows:

(11) $$\Phi_{ij}\left(x\right)=\frac{1}{{\left(2\pi \right)}^{\frac{1}{2}}\sigma^{d}}e^{-\frac{{\left(X-x_{ij}\right)}^{T}\left(X-x_{ij}\right)}{\sigma^{2}}}$$

where $X={\left[x_{1},x_{2},\ldots ,x_{n}\right]}^{T},n=1,2,\ldots ,l$. *d* is the input feature dimension, and *l* is all training types. $x_{ij}$ represents the *j*th data of the *i*th neuron. σ represents the smoothing factor. The summation layer averages the output weights of neurons belonging to the same type of pattern layer, and the results can be calculated by

(12) $$v_{i}=\frac{\sum_{j=1}^{L}\Phi_{ij}}{L}$$

where $v_{i}$ represents the output of type *i*, and *L* represents the number of type *i* neurons. The output layer is responsible for outputting the highest scoring category in the summation layer, and the output is as follows:

(13) $$Type\left(v_{i}\right)=arg\;\max\left(v_{i}\right)$$

**Figure 1:**
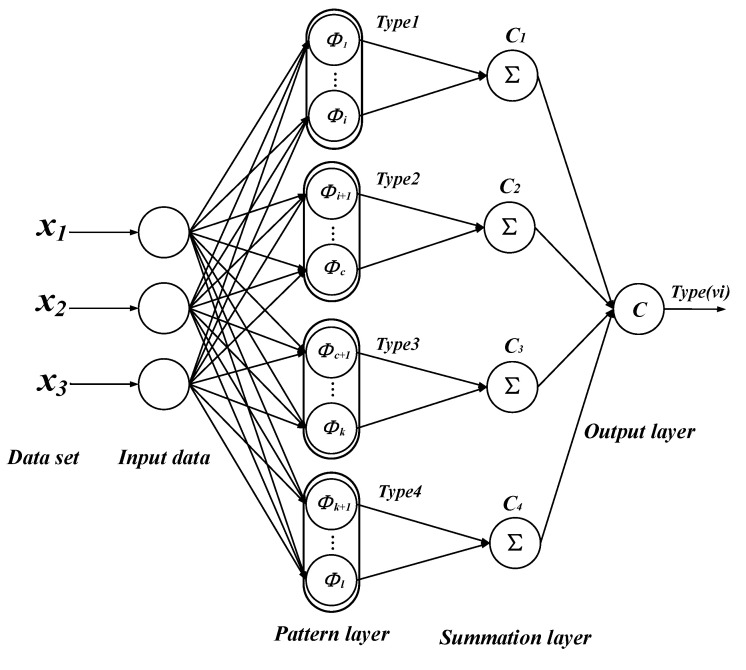
Probabilistic neural network structure diagram.

In the topology of PNN: the number of input layers is the number of sample features, the number of neurons in the pattern layer is the number of input sample vectors, and the number of neurons in the summation layer is the number of sample categories. Therefore, if we assume a pattern recognition task with four categories of samples, a variable number of samples in each category, and a three-dimensional feature dimension for each sample, we can draw the network structure as shown in Figure 1.

### 2.5. The Proposed ISSA-PNN Model

**Figure 2:**
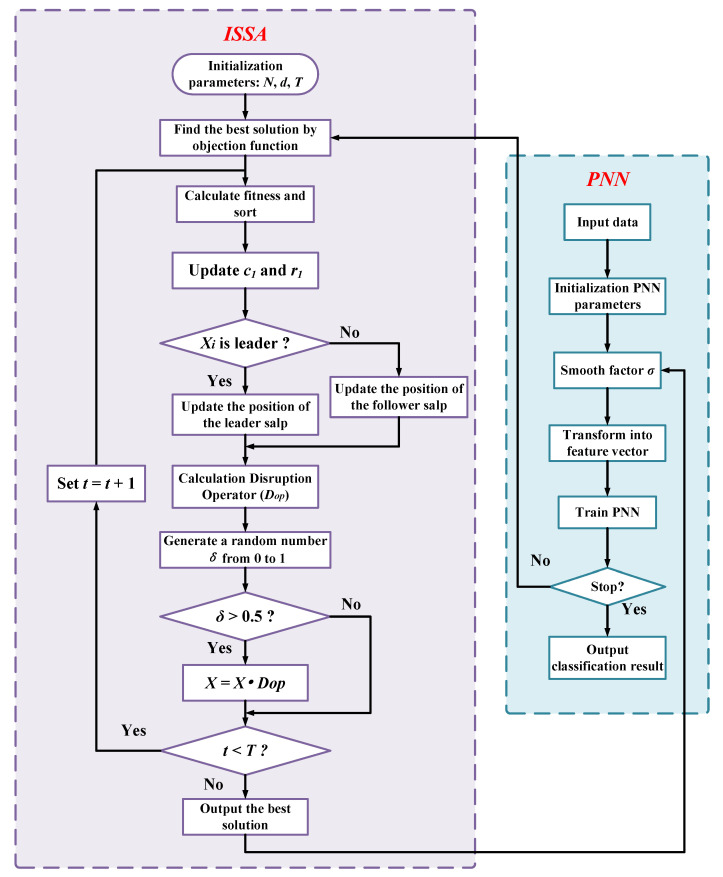
The diagram of the proposed ISSA-based PNN for fault diagnostics.

The classification performance of a PNN is easily affected by the smoothing factor σ. Choosing an overly large or overly small value of σ will make the network converge too quickly and thus fail to find the optimal solution, making the diagnostic classification accuracy drop significantly. To this end, we use the hybrid algorithm ISSA to find the most suitable σ to improve the classification performance of the network, to establish a practical performance, high accuracy, and reliable ISSA-PNN fault diagnosis model, and the optimization process of ISSA on PNN can be represented by Figure 2.

To this end, we used the hybrid algorithm ISSA to find the most suitable σ to improve the classification performance of the network, to establish the ISSA-PNN fault diagnosis model with good practical performance, high accuracy, and reliability. Moreover, the optimization process of ISSA-PNN can be represented by Figure 2.

The main specific steps of the ISSA-PNN fault diagnosis model are shown below.

- **Step 1:** The pre-processed DGA data are input into PNN randomly, and the parameters are initialized.
- **Step 2:** The initial parameters of ISSA are set: population size *N*; dimension *d*; and the maximum number of iterations *T*. Moreover, the population position of ISSA is initialized by Equation (1), and each salp individual represents a set of smoothing factors σ.
- **Step 3:** The salp group’s fitness values within the population were calculated and ranked. In this paper, the mean square error is set as the fitness function, as shown in Equation (14).
  (14) $$f\left(x\right)=\frac{1}{N}\sum_{i=1}^{N}{\left(Y_{i}-O_{i}\right)}^{2}$$
- **Step 4:** The one with the best adaptation is considered as the current food position. Among the remaining N−1 salps, the salps with the top half of adaptation are considered as the leader, and the rest of the salps are considered as followers.
- **Step 5:** Update $r_{1}$ and $c_{1}$ according to Equation (3) and Equation (8), respectively.
- **Step 6:** Update the leader position by Equation (2) and the follower position by Equation (7).
- **Step 7:** Calculate $D_{op}$ by Equation (9) and generate a random number $\delta_{0}$. If $\delta_{0}$ is greater than 0.5, diversify the salp population, according to Equation (10).
- **Step 8:** If the current number of iterations reaches the maximum number of iterations, then proceed to the next step—otherwise, return to Step 5.
- **Step 9:** Input ISSA optimized smoothing factor into PNN to obtain a better performance PNN model and the input test set data into PNN to obtain the best diagnostic results.

## 3. Implementation and Experiment Setup

### 3.1. Model Implementation

**Figure 3:**
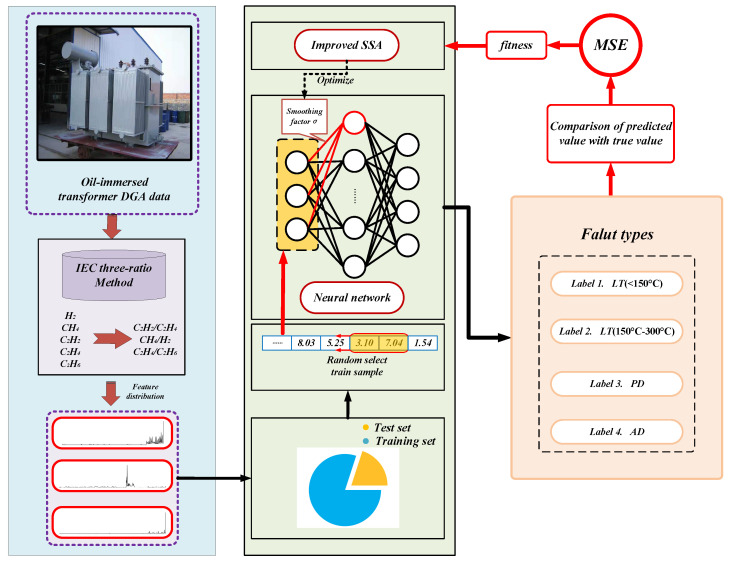
The implemented framework of the power transformer fault diagnosis.

The implementation framework of the proposed ISSA-PNN model for power transformer fault diagnosis is shown in Figure 3. As can be seen from the figure, the implementation of the proposed model is divided into three parts: data collection and processing, the training of the neural network, and the testing and evaluation of the network model. Firstly, some dissolved gas content of transformer oil is collected from the smart sensors inside the oil-immersed transformer as DGA data, and then the collected DGA data are pre-processed and filtered using an IEC three-ratio method. A random selection of 80% of the pre-processed DGA data are input into the ISSA-PNN model for training and optimization. The remaining data are used for testing and evaluating the performance of the diagnostic model.

In this paper, we focused on four types of oil-immersed power transformer faults to train and test the diagnostic models, namely low temperature and overheating (LT) (<150 °C); low temperature and overheating (LT) (150–300 °C); partial discharge (PD); and arc discharge (AD). Table 1 shows some real data for judging the fault types of oil-immersed power transformers by the DGA method from the power supply companies (PSCs) in some provinces of China. In addition, since the proposed model was based on a probabilistic neural network, each fault type will be coded in the form as shown in Table 2.

### 3.2. Data Collection and Pre-Processing

To ensure the validity of the experiment and reduce the influence of temperature, humidity, transformer model, and other parameters on the experimental data, we deliberately collected several groups of sensor data of various gases dissolved in oil-immersed transformer oil from Jiangxi Power Supply Company and transformer factory as experimental data samples. For dissolved gas analysis, we selected the volume fraction of some gases (C${}_{2}$H${}_{2}$, C${}_{2}$H${}_{4}$, CH${}_{4}$, H${}_{2}$, C${}_{2}$H${}_{6}$) dissolved in transformer oil as the primary basis for transformer fault type judgment.

After excluding some noisy data and incomplete data samples and processing them by the IEC three-ratio method, 555 valid characteristic gas data samples were obtained, including 361 sets of low-temperature overheating (LT) (<150 °C), 40 sets of low-temperature overheating (LT) (150–300 °C), 65 sets of partial discharge (PD), and 89 sets of arc discharge (AD). Among them, 444 sets of data were used as training samples, and 111 sets of data were used as test samples. Some of the data samples are shown in Table 3.

**Figure 4:**
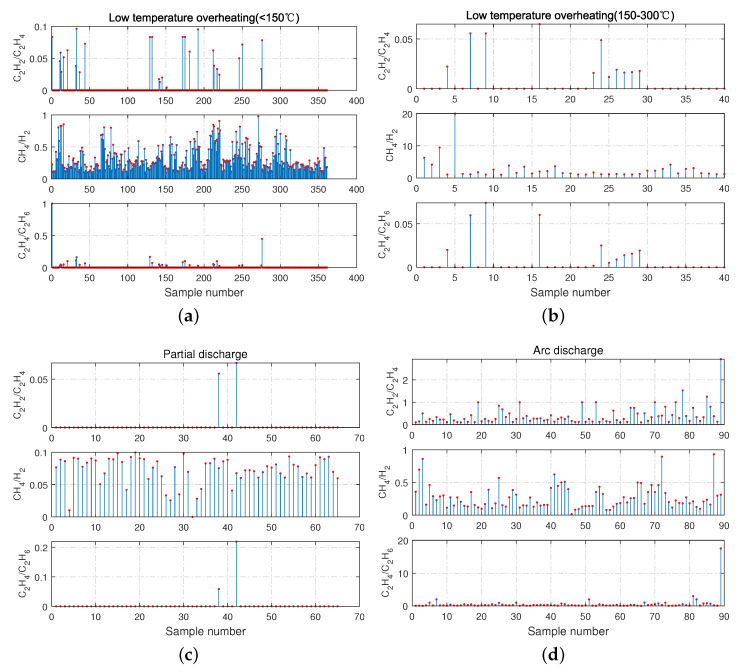
The Dissolved gas data distribution of three-ratio for four fault types. (**a**–**d**), in the order of low temperature overheating (<150 ℃), low temperature overheating (150–300 ℃), partial discharge, and arc discharge.

The distribution of dissolved gas data for the four-fault types after pre-processing is shown in Figure 4, and it can be seen that the distribution of C${}_{2}$H${}_{2}$/C${}_{2}$H${}_{4}$, CH${}_{4}$/H${}_{2}$, and C${}_{2}$H${}_{4}$/C${}_{2}$H${}_{6}$ gas ratios is with apparent differences. Although the data samples of low-temperature overheating (<150 ℃) fault are large, the data distribution is the most complicated, which is a problematic point in fault diagnosis.

### 3.3. Performance Evaluation

For validating the performance of the proposed model in power transformer fault diagnosis, we compared ISSA-PNN with the conventional PNN model and the PNN model optimized by particle swarm optimization (PSO), seagull optimization algorithm (SOA), bat algorithm (BA), multi-verse optimizer (MVO), and salp swarm algorithm (SSA), respectively. Secondly, we compared with other hybrid back propagation neural network (BP) models mentioned in the similar research literature [35,36], including BA-BP, cuckoo search (CS)-BP, genetic algorithm (GA)-BP. Moreover, we compared with some standard classical diagnostic methods for comparison, such as the IEC ratio method, the support vector machine (SVM), and the multi-layer perceptron (MLP). The parameter settings of different optimization methods are detailed in Table 4.

To effectively demonstrate the effectiveness of the proposed model, this paper will be evaluated by accuracy and F1-score. The confusion matrix is an essential criterion for the classification model. As shown in Table 5, it contains four values, which are a true positive (TP), true negative (TN), false positive (FP), and false negative (FN).

Precision is the ratio of the number of positives correctly predicted to the number predicted to be positive, as shown in Equation (15):

(15) $$Precision=\frac{TP}{TP+FP}$$

Recall is the ratio of the number of positives correctly predicted to the number of actual positive examples, as shown in Equation (16):

(16) $$Recall=\frac{TP}{TP+FN}$$

The other two crucial evaluation metrics can be obtained from Table 5, i.e., accuracy and F-score, calculated as follows:

(17) $$Accuracy=\frac{TP+TN}{TN+TP+FN+FP}$$

(18) $$F-score=\left(1+\beta^{2}\right)\frac{Precision\times Recall}{\left(\beta^{2}\cdot Precision\right)+Recall}$$

When β is equal to 1, this evaluation metric is called the balanced F-score (F1-score), indicating that recalls and precisions are weighed on the same footing for consideration. In this paper, β is 1.

## 4. The Experimental Results

We performed simulation training on the MATLAB platform with the same test set and training set. We compared it with five other modified PNN methods and traditional PNN, and the results of each failure and average accuracy are shown in Table 6. It can be seen that the average accuracy of ISSA-PNN is 99.65%, which is higher than the other methods: SSA-PNN 97.37%; MVO-PNN 97.02%; BA-PNN 96.52%; SOA-PNN 95.80%; PSO-PNN 94.49%; and PNN 86.70%. In addition, the ISSA-PNN method only has error cases at LT (<150 °C) with an accuracy of 98.59%.

Moreover, to further prove the excellence of the proposed method, we compared it with the excellent methods proposed by other researchers and some classical methods. The comparison results are shown in Table 7. It can be seen that the average accuracy of ISSA-PNN is also superior to other methods, although ISSA-PNN (98.59%) is inferior to BA-BP (99.06%) and GA-BP (99.06%) methods in LT (<150 °C) faults. However, the remaining three fault types’ performance is much better than BA-BP and GA-BP, so the combined performance of ISSA-PNN is still the best, proving the excellent performance of the proposed method in a power transformer fault diagnosis.

**Figure 5:**
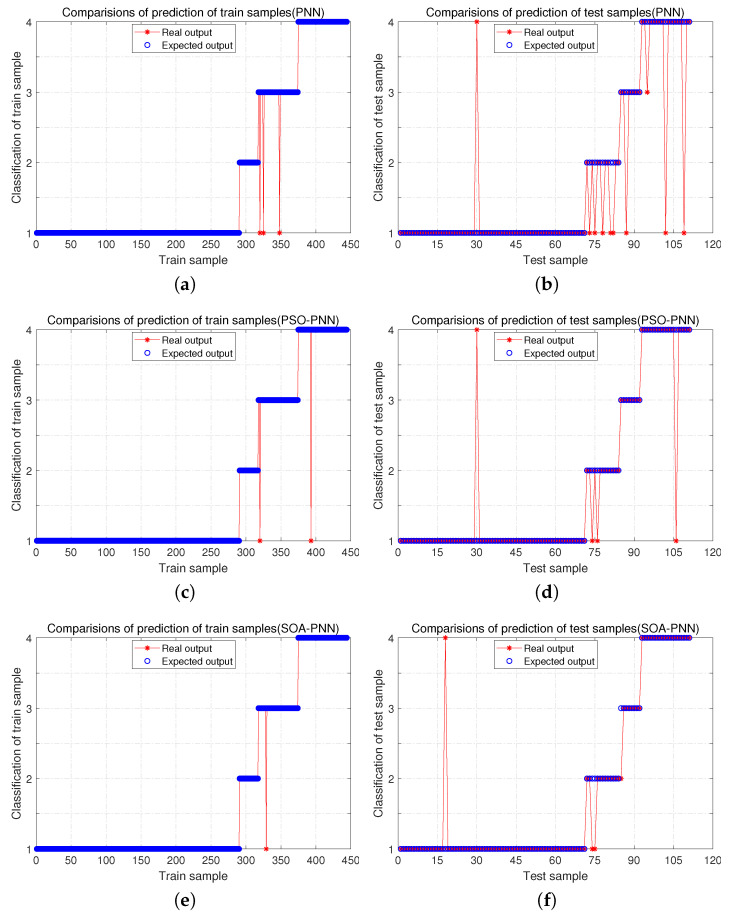

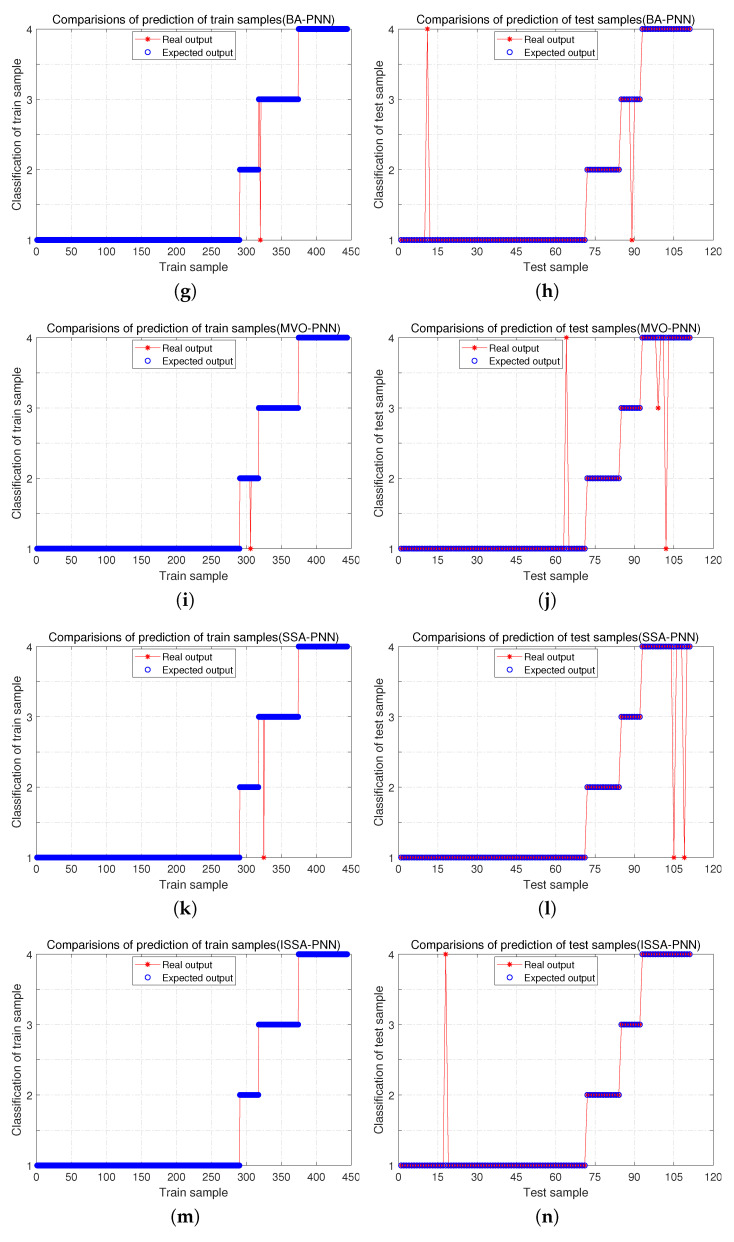
The classification results of different methods.

Figure 5 shows the classification results of different methods on data samples after training, where subgraphs a, c, e, g, i, k, m are the classification results of training samples, and subgraphs b, d, f, h, j, l, n are the classification results of test samples.

**Figure 6:**
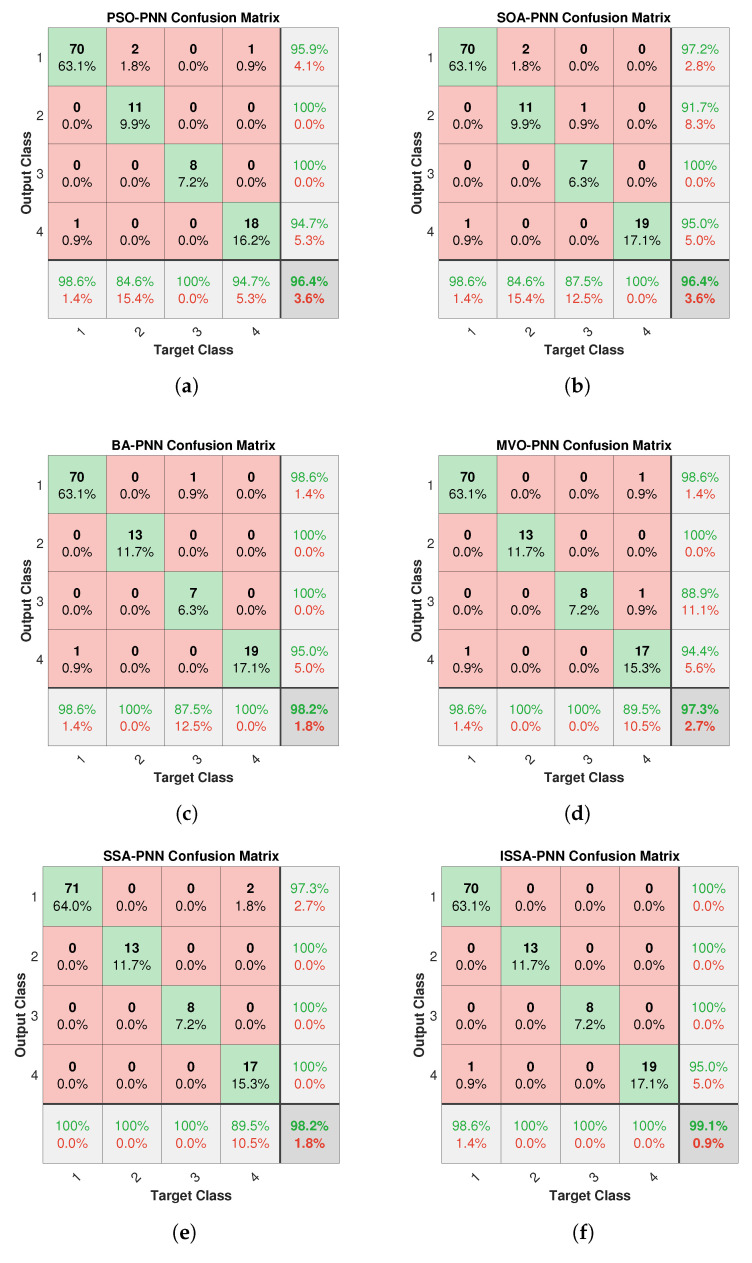
Confusion matrix for different methods.

The confusion matrix is one of the crucial tools for evaluating classification models. We plotted the confusion matrix of various methods to compare the performance of each method, as shown in Figure 6. The target classes 1, 2, 3, and 4 in the subgraphs refer to LT (<150 °C), LT (150–300 °C), PD, and AD, respectively. In addition, the rightmost column of the subgraph is the precision (or positive predictive value), and the bottom row is the recall (or true positive rate). It is worth noting that the lower right cell indicates the overall accuracy, which is different from the average accuracy in Table 6 and Table 7.

According to the confusion matrix in Figure 6, the F1-score corresponding to each method can be calculated, which is one of the critical indicators for evaluating classification models, and the calculation results are shown in Table 8. It can be seen from Table 8 that the F1-score of the four-fault types of ISSA-PNN are 99.29%; 100.00%; 100.00%; 97.44%; and the Marco F1-score is 99.18%, which are higher than the other method models.

**Figure 7:**
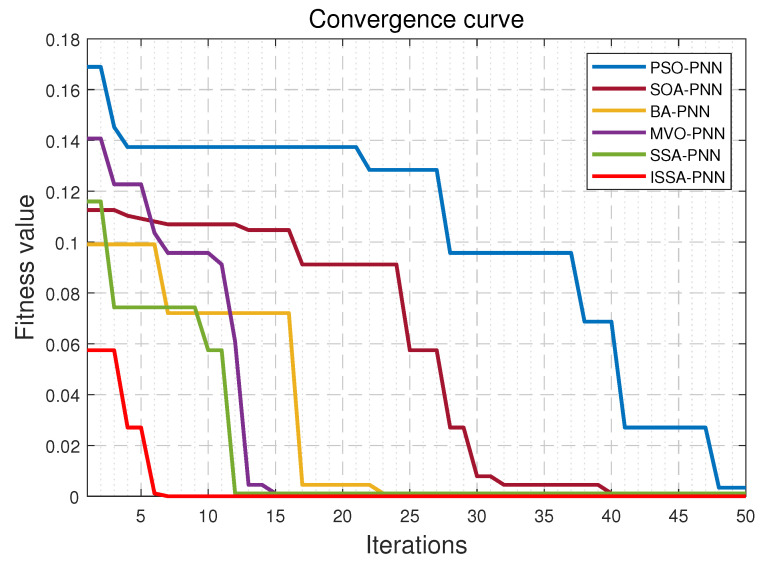
The fitness value curve of different optimization methods.

The comparison of MSE for different methods is shown in Table 9. Figure 7 shows the change curve of the fitness of different algorithms in the optimization process for PNN, which can well show the optimization process of various algorithms for comparison.

## 5. Discussion

As can be seen from Table 6 and Table 7 regarding the comparison in terms of accuracy, ISSA-PNN has the best overall performance among all four faults, both compared with various optimized PNN methods and with other excellent improved neural network methods, and is only slightly inferior to BA-BP and GA-BP in LT (<150 °C) faults. Except for LT (<150 °C) fault, ISSA-PNN is clearly the best in the remaining three faults and the final average accuracy, especially in AD fault diagnosis where the accuracy is generally low, ISSA-PNNN still maintains 100% accuracy.

Figure 5 shows that the performance of other algorithms in training samples and test samples is not ideal, and there are generally multiple errors. Many algorithms perform well during training, and once they switch to test samples for model testing, there is a sudden increase in errors, which indicates that these algorithms are less robust to the point that they fall into overfitting during training. From Figure 5m,n, it can be seen that ISSA-PNN has no error in training samples, and the results are ideal. In the test samples, only errors are generated in the first type of faults, and the classification of the remaining faults is correct. This indicates that the proposed method not only has good accuracy but also has strong robustness and does not easily fall into overfitting during the training process which degrades the model performance.

In Table 8, the F1-score of the ISSA-PNN method is the highest, consistent with the accuracy assessment results, indicating that the accuracy assessment results have high reliability. It was also fully demonstrated that ISSA-PNN has the best comprehensive performance in power transformer fault diagnosis and can meet various engineering needs in terms of precision and recall.

In Table 9, ISSA-PNN performs the best in training with the MSE of 0. Moreover, ISSA-PNN does not have the same as the GA-BP method: the MSE during training is very low, second only to ISSA-PNN (0.005), but it falls into overfitting, and its performance during testing is not satisfactory (only 0.19030). The result indicates that ISSA-PNN can learn the internal laws implied by the data more quickly and effectively, has excellent generalization ability and self-regulation ability, and can better cope with the interference of various random noises in practical applications.

As can be seen in Figure 7, for ISSA-PNN, its fitness starts decreasing at the fourth iteration, and falls into the local optimum at the fifth iteration, and then jumps out of the local optimum at the sixth iteration to continue the optimization search, and finally reaches the global optimum at the seventh iteration. In contrast, other optimization algorithms take several iterations to struggle to jump out of the local optimum. Most of the decreases in the fitness of each iteration are not as large as those of the ISSA algorithm. It can be seen that compared with the original SSA algorithm and other algorithms for PNN optimization, ISSA has a robust global search capability and can quickly jump out of the local optimum without getting trapped in it and find the global optimum solution quickly. The rapid and efficient convergence process of ISSA-PNN fully shows its practicality in engineering applications. It is worth noting that the initial fitness value of the ISSA algorithm model is smaller than other algorithm models, which indicates that it is less susceptible to some initial noise and has better stability and anti-interference ability.

These results all reflect that the ISSA-PNN method is superior to other methods with better diagnostic accuracy, robustness, and generalization capability. Therefore, the proposed method has high applicability, reliability, and practicality in the field of oil-immersed transformer fault diagnosis.

## 6. Conclusions

In this paper, the PNN and DGA methods were combined to establish a basic fault diagnosis model. Then, a bio-inspired optimization algorithm was introduced to optimize the smoothing factor, which is an important parameter of PNN, to improve the performance of the fault diagnosis model. Meanwhile, we also improved the traditional SSA by introducing the SCA algorithm and disruption operator ($D_{op}$) to enhance the search capability of the traditional SSA algorithm, which enables the solution space to be sufficiently searched to prevent falling into the local optimum. Furthermore, the search time was also reduced to meet the practical engineering requirements. We compared the proposed method with other classical and excellent models using real data collected from sensors installed inside the transformers and evaluated them by multiple dimensions and multiple metrics. The experimental results show that the ISSA-PNN method has better diagnostic performance in power transformer fault diagnosis, can overcome some initial error interference, does not easily fall into overfitting, and has good robustness and accuracy.

## Figures and Tables

**Table 1:** Some real data from power supply companies in some provinces of China diagnosing power transformer fault types by DGA method.

Fault Type	Dissolved Gas (µL/L)					Sources
	CH${}_{4}$	C${}_{2}$H${}_{2}$	C${}_{2}$H${}_{4}$	C${}_{2}$H${}_{6}$	TH	
LT (<150 ℃)	83	53	13	1.2	150.2	Jiujiang PSC
LT (150–300 ℃)	6.5	98	16	1.5	122	Fuzhou PSC
LT (150–300 ℃)	193	191	28	16	428	Yingtan PSC
LT (150–300 ℃)	12	46	11	1.8	70.8	Nanchang PSC
LT (150–300 ℃)	3.5	31	8.2	1	43.7	Yichun PSC
AD	61	307	105	6	479	Yingtan PSC

**Table 2:** Coding format for different fault types.

Fault Type	LT (<150 ℃)	LT (150–300 ℃)	PD	AD
Coding format	1	0	0	0
	0	1	0	0
	0	0	1	0
	0	0	0	1

**Table 3:** Partial sample data.

Dissolved Gas (µL/L)			Fault Type
C${}_{2}$H${}_{2}$/C${}_{2}$H${}_{4}$	CH${}_{4}$/H${}_{2}$	C${}_{2}$H${}_{4}$/C${}_{2}$H${}_{6}$	
0.05172	0.85455	0.04839	LT (<150 ℃)
0	0.17529	0	LT (<150 ℃)
0.0625	0.15517	0.1	LT (<150 ℃)
0.01899	1.21828	0.00885	LT (150–300 ℃)
0.01613	1.125	0.01389	LT (150–300 ℃)
0.01667	1.08108	0.01563	LT (150–300 ℃)
0.05556	0.07524	0.05882	PD
0	0.07059	0	PD
0.06667	0.06754	0.21910	PD
0.01613	1.12500	0.01389	PD
0.01667	1.08108	0.01563	PD
0.01786	1.23188	0.01923	PD
0.375	0.45882	0.75	AD
0.4	0.89361	0.28571	AD
0.8	0.33928	1	AD
0.25	0.32323	0.33333	AD
0.14844	0.07836	0.14394	AD

**Table 4:** Parameter setting of various methods.

Methods	Parameters Settings
PSO-PNN	c1 = c2 = 1.49445
SOA-PNN	NP = 10, T = 50
BA-PNN	NP = 20, A = 0.5, r = 0.5
MVO-PNN	NP = 10, T = 50
SSA-PNN	NP = 6, T = 50
ISSA-PNN	NP = 3, T = 10, δ = 0.5
BA-BP	NP = 20, A = 0.5, r = 0.5
CS-BP	NP = 20, Pa = 0.25
GA-BP	NP = 20, Pm = 0.01, Px = 0.7

**Table 5:** Confusion matrix.

Actual Class	Predicted Class	
	Positive	Negative
Positive	True positive (TP)	False negative (FN)
Negative	False positive (FP)	True negative (TN)

**Table 6:** Accuracy comparison of different optimized PNN methods.

Fault Type	Accuracy (%)						
	ISSA-PNN	SSA-PNN	MVO-PNN	BA-PNN	SOA-PNN	PSO-PNN	PNN
LT (<150 ℃)	98.59	100.00	98.59	98.59	98.59	98.59	95.77
LT (150–300 ℃)	100.00	100.00	100.00	100.00	84.62	84.62	61.54
PD	100.00	100.00	100.00	87.50	100.00	100.00	100.00
AD	100.00	89.47	89.47	100.00	100.00	94.74	89.47
Average	99.65	97.37	97.02	96.52	95.80	94.49	86.70

**Table 7:** Accuracy comparison of different methods.

Fault Type	Accuracy (%)						
	ISSA-PNN	BA-BP	CS-BP	GA-BP	MLP	SVM	IEC
LT (<150 ℃)	98.59	99.06	94.34	99.06	91.55	84.51	97.17
LT (150–300 ℃)	100.00	92.31	100.00	92.31	100.00	92.31	100.00
PD	100.00	100.00	100.00	100.00	62.50	75.00	7.14
AD	100.00	95.45	90.91	81.82	78.95	68.42	100.00
Average	99.65	96.71	96.31	93.30	83.25	80.06	76.08

**Table 8:** Accuracy comparison of different methods.

Fault Type	F1-Score (%)						
	ISSA-PNN	SSA-PNN	MVO-PNN	BA-PNN	SOA-PNN	PSO-PNN	PNN
LT (<150 ℃)	99.29	98.61	98.59	98.61	97.90	97.22	93.79
LT (150–300 ℃)	100.00	100.00	100.00	100.00	91.67	91.67	76.19
PD	100.00	100.00	94.12	93.33	100.00	100.00	88.89
AD	97.44	94.44	91.89	97.44	97.44	94.74	89.47
Marco F1-score	99.18	98.26	96.15	97.35	96.75	95.91	87.09

**Table 9:** Comparison of sample errors.

Methods	MSE of Training	MSE of Test
ISSA-PNN	0.00000	0.08108
SOA-PNN	0.00901	0.10910
BA-PNN	0.00901	0.11712
MVO-PNN	0.00225	0.17117
SSA-PNN	0.00901	0.16216
PSO-PNN	0.02928	0.18018
BA-BP	0.02500	0.13100
CS-BP	0.00750	0.15480
GA-BP	0.00500	0.19030
PNN	0.03703	0.33333
MLP	0.04277	0.38013
SVM	0.04344	0.41231
IEC	0.05625	0.46770

## Data Availability

Not applicable.

## Abbreviations

The following abbreviations are used in this manuscript:

ANN	Artificial neural network
AD	Arc discharge
BA	Bat algorithm
BP	Back-propagation
BPNN	Back-propagation neural network
CS	Cuckoo search
CVA	Common vector approach
D${}_{op}$	Disruption operator
DADDNN	Dynamic Adam and dropout-based deep neural network
DBN	Deep belief network
DGA	Dissolved gas analysis
GA	Genetic algorithm
GWO	Gray wolf optimization
IEC	International electrotechnical commission
ISSA	Improved salp swarm algorithm
KNN	K-nearest neighbor
LT	Low temperature and overheating
MLP	Multi-layer perceptron
MSE	Mean square error
MVO	Multi-verse optimizer
PD	Partial discharge
PNN	Probabilistic neural network
PSC	Power supply companies
PSO	Particle swarm optimization
SCA	Sine cosine algorithm;
SD	Standard deviation
SOA	Seagull optimization algorithm
SSA	Salp swarm algorithm
SVM	Support vector machine
σ	Smoothing factor
